# Giant Chest Wall Metastasis of Rectal Adenocarcinoma with Multistructural Involvement

**DOI:** 10.3390/jcm15072654

**Published:** 2026-03-31

**Authors:** Dawid Murawa, Joanna Jaśkiewicz, Zachariasz Rytelewski, Aleksander Murawa, Paula Dobosz, Tomasz Grodzki, Paweł Zieliński

**Affiliations:** 1 Collegium Medicum, University of Zielona Góra, 65-046 Zielona Góra, Poland; 2 Department of General and Oncological Surgery, The Regional Hospital in Poznan, The Greater Poland Specialist Center, 60-479 Poznan, Poland; 3 Faculty of Medicine, Poznan University of Medical Sciences, 60-355 Poznan, Poland; 4 Department of Patomorphology, Poznan University of Medical Sciences, 60-355 Poznan, Poland; 5 Department of Thoracic Surgery and Transplantology, Pomeranian Medical University in Szczecin, 70-891 Szczecin, Poland

**Keywords:** rectal adenocarcinoma, metastasis, rectal cancer, pleura metastasis, rib metastasis, case report, HITHOC

## Abstract

**Introduction and Importance**: Colorectal adenocarcinoma typically metastasizes to the liver and lungs, with pleural, breast, or osseous involvement being exceedingly rare. Here, we report an unusual case of rectal adenocarcinoma metastasizing to the chest wall with simultaneous involvement of the lung, pleura, ribs, and subcutaneous breast tissue, forming a dominant giant metastasis (25 × 18 × 16 cm) accompanied by additional satellite lesions between the ribs and pectoral muscles, as well as intrapulmonary nodules. **Presentation of case**: The patient underwent radical resection including rib excision, followed by hyperthermic intrathoracic chemotherapy (HITHOC) with mitomycin. Chest wall integrity was restored using a synthetic mesh and titanium plating, ensuring both oncologic clearance and structural stability. Multimodal therapy also included neoadjuvant chemotherapy with bevacizumab, which was continued postoperatively. **Clinical discussion**: This case underscores the critical role of a multidisciplinary strategy in managing rare and aggressive metastatic patterns of colorectal cancer. In selected patients, a combination of systemic therapy, extensive surgical resection, advanced reconstruction, and regional chemotherapy may offer the potential for short-term local disease control. **Conclusions:** The radical excision of the giant tumour enabled continuation of systemic therapy under the national drug programme, was associated with short-term local control, and improved the patient’s quality of life.

## 1. Introduction

Rectal adenocarcinoma exhibits tendency for distant metastasis, most commonly involving the liver (~70%), followed by the lungs (≈30–50%); less frequent sites include bone, the central nervous system, and other extra-abdominal locations [1,2,3]. Metastatic involvement of the pleura, chest wall soft tissues (including breast/subcutaneous tissue), and ribs is extremely rare in rectal cancer; most reports are isolated case reports or very small series [4,5,6,7,8,9,10,11,12,13,14,15]. For example, literature reviews show perhaps only a handful of documented cases of colorectal adenocarcinoma metastasizing to the chest wall with extension into breast or subcutaneous breast tissue [4,5,6,7,8,9,10,11,12,13,14,15]. Therefore, we wished to present the therapeutic approach we applied, as it may assist in identifying an effective management strategy for this type of metastasis. The management of such rare and complex cases often necessitates an experimental therapeutic approach, particularly in the absence of well-established treatment guidelines. Furthermore, similar cases may occur in other medical centres, highlighting the potential relevance of this experience.

We herein report the case of a 51-year-old male who, at the time of chest wall resection, had previously undergone radical surgery for primary rectal cancer and liver and lung metastasectomies, and subsequently developed a massive chest wall metastasis invading the lung, pleura, ribs, and subcutaneous breast tissue. He underwent a neoadjuvant regimen including bevacizumab, en bloc resection with HITHOC (hyperthermic intrathoracic chemotherapy) using mitomycin, chest wall reconstruction with synthetic mesh and metal plating. Postoperatively, he has continued systemic therapy and remains alive at the time of writing.

### 1.1. Additional Information

This case report has been reported in line with the SCARE checklist [16].

In accordance with the TITAN-AI guidelines, all the authors declare that no artificial intelligence tools were used in the preparation, writing, or editing of this manuscript [17].

### 1.2. Informed Consent

Written informed consent was obtained from the patient for publication of this case report and any accompanying images.

### 1.3. Ethical Approval

Ethical approval was not required for this single-patient case report, as it does not meet the definition of medical research according to Polish national legislation and did not require review by the institutional ethics committee.

### 1.4. Research Registration

Research registration was not applicable for this single-patient case report.

## 2. Case Presentation

**Figure 1:**
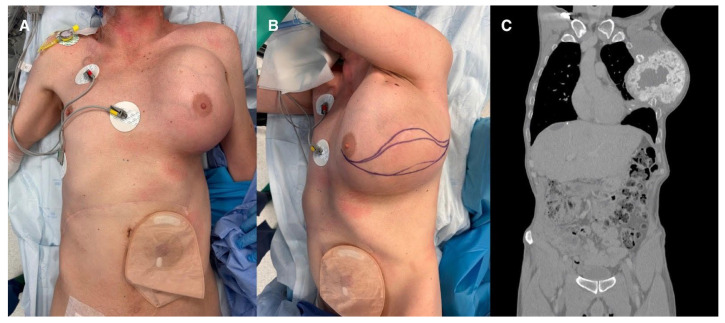
(**A**). Patient before surgery anterior view. (**B**). Patient before surgery lateral view. (**C**). Patient before surgery CT scan anterior view (This figure should be printed in colour).

A 51-year-old male was referred to our department with a very large metastatic rectal adenocarcinoma involving the chest wall, with infiltration of the subcutaneous tissue, ribs, and pleura. He reported no significant complaints at the time of assessment and denied any history of chronic illnesses or allergies. The patient had a history of anterior resection of the rectum, resection of hepatic metastases, and wedge resection of a metastatic lesion in the left upper lung lobe, as well as chemotherapy with FOLFOX4/LF4. Following the detection of the lesion on 14 November 2024, systemic treatment combined with bevacizumab was initiated, but it proved ineffective with respect to the chest-wall and pulmonary involvement, as subsequent CT imaging demonstrated progression (Figure 1). No other distant metastases or local recurrence at the primary site were identified. The case was reviewed by a multidisciplinary team (MDT) including a thoracic surgeon, surgical oncologist, radiologist, and anaesthesiologist. Radiological assessment determined the extent of chest wall and pulmonary involvement and confirmed technical resectability. The anaesthesiology team evaluated the patient’s suitability for major thoracic surgery and general anaesthesia, considering the anticipated complexity and duration. Surgical planning also included strategies for chest wall reconstruction following extensive resection. Given the large metastatic lesion from rectal adenocarcinoma involving the chest wall and adjacent lung, the MDT concluded that en bloc resection offered the best chance for local disease control.

Contrast-enhanced computed tomography (CT) demonstrated a dominant calcified chest wall metastasis causing bony destruction and a pathological fracture of the left fourth rib, with minor invasion of the fifth rib. The dominant lesion measured 82 × 61 mm. In addition to the main mass, two smaller satellite lesions were identified between the ribs and the pectoral muscles. The total tumour complex measured approximately 25 × 18 × 16 cm. The medial lesion showed calcifications, whereas the lateral lesion exhibited areas of necrosis.

There was also pleural effusion in the left hemithorax with a maximal thickness of 17 mm. Additional metastatic disease included a 22 mm lesion in segments LS1 and LS2, a 7 mm subpleural nodule in LS6 (suspicious for metastasis), and a 43 × 25 mm soft-tissue thickening along the fissure in LS5, with a smaller 7 mm nodule located inferiorly. In the right lung, scattered nodules up to 6 mm in size were observed. No other neoplastic lesions were identified.

**Figure 2:**
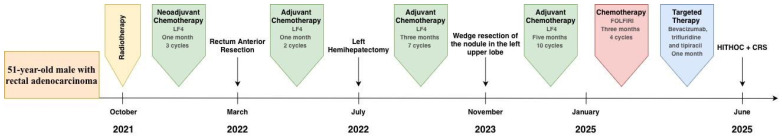
Clinical history (This figure should be printed in colour).

The choice and modification of systemic chemotherapy were made by the treating oncologist and the multidisciplinary oncology team at an external oncology centre. The transition from LF4-based treatment to FOLFIRI in 2025 reflected oncological decision-making based on disease progression and prior treatment exposure. A complete clinical history of our patient has been depicted on Figure 2.

The surgery was performed 5 June 2025 by two experienced thoracic surgeons (with over 30 years of continuous practice) and a surgeon specialized in general surgery and surgical oncology at a regional (voivodeship) hospital. The patient received perioperative antibiotic prophylaxis with piperacillin. During tumour dissection, both the lesion in the chest wall and the pulmonary lesions were assessed as resectable. The approximately 20 × 30 cm tumour was carefully dissected from the subcutaneous tissue, partially separating the pectoralis major and latissimus dorsi muscles down to the rib cage. The pleural cavity was opened, and the fourth rib was transected near its parasternal portion while the fifth rib was divided approximately 4 cm from its sternal attachment. The tumour penetrated the pleural cavity and was partially adherent to mediastinal fat and the upper lobe of the left lung, from which an additional nodule was excised using a stapler. The mass was resected en bloc with involved rib segments. Mechanical staplers were used for pulmonary resection while advanced energy devices (LigaSure) and standard electrosurgical instruments such as monopolar electrosurgery and bipolar forceps, were employed for soft tissue dissection and haemostasis.

Intraoperative HITHOC was then performed with mitomycin C at a dose of 40 mg at 42 °C for 60 min. The dose of mitomycin C in HITHOC was calculated based on body surface area 35 mg/m^2^. Notably, this represents an off-label application of HITHOC used as a regional adjunct after macroscopic cytoreduction in the absence of guideline-directed treatment options for pleural metastases from colorectal cancer. The primary aim of this intervention was to improve loco-regional disease control following extensive pleural involvement.

As no standardized HITHOC protocol exists for colorectal pleural metastases, the choice of mitomycin C was pragmatic and based on institutional experience. Importantly, we do not claim evidence-based superiority of this regimen in this setting, and the procedure should be considered experimental.

**Figure 3:**
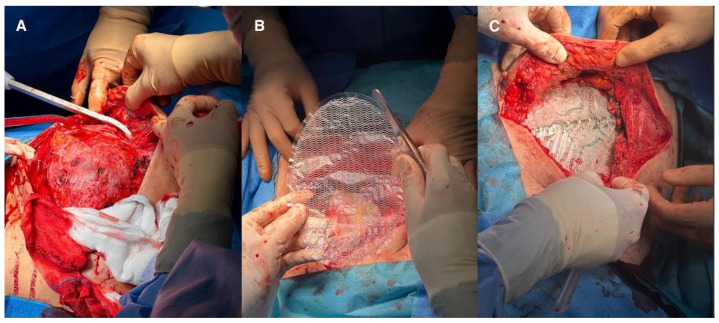
(**A**). Dissection of the tumour from the skin and subcutaneous tissue. (**B**). Polypropylene mesh. (**C**). Post-reconstruction status of the chest wall. (This figure should be printed in colour).

**Figure 4:**
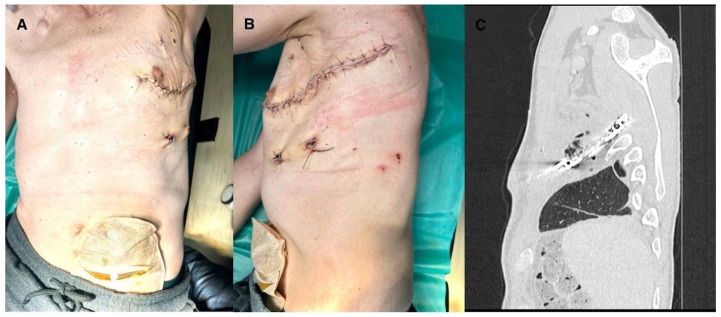
(**A**). Patient after surgery anterior view. (**B**). Patient after surgery lateral view. (**C**). Patient after surgery CT scan lateral view (This figure should be printed in colour).

Reconstruction of the chest wall defect was achieved using a 19 cm metal bridging plate (Narrow plate, ChM system; ChM sp. z o.o., Juchnowiec Kościelny, Poland) and a polypropylene mesh (2P-PCMC ClearMesh Composite; Dipromed S.r.l., San Mauro Torinese, Italy). (Figure 3) The wound was closed in layers and covered with a negative-pressure dressing. The total operative time for the chest wall metastasis resection was 4 h and 38 min. The amount of intraoperative blood loss was 500 mL. Postoperative recovery proceeded without complications. Results of the closure are shown on Figure 4. On the anaesthesiological side, operation proceeded smoothly, without any significant disruptions. The final histopathological examination confirmed an R0 resection, with microscopically negative margins.

The follow-up included a chest X-Ray and ultrasonography after two weeks from the moment of admission. Blood tests assessing full blood count and inflammatory markers were repeated after one week, two weeks, and then monthly. CT scans of the chest and abdomen were performed one month after hospital discharge and repeated three months later. Both imaging studies and laboratory tests demonstrated stabilisation of the patient’s condition. The follow-up protocol, including CT imaging, was performed in accordance with institutional policy for patients undergoing extensive surgical procedures.

The documented postoperative follow-up period was approximately 4.5 months from the date of surgery (5 June 2025 to 24 October 2025). No radiological signs of local recurrence or distant progression were observed. On 24 October 2025 (second follow-up CT), no definitive evidence of recurrence was identified. However, small suspected lesions were described: a subpleural lesion in left segment 6 (LS6), measuring 8 mm, nodular changes at the level of the 4th and 5th rib stumps, measuring approximately 11 mm and 17 mm, respectively. These findings were classified as radiologically suspected but not confirmed recurrence, and further surveillance imaging was recommended. At short-term follow-up, no confirmed recurrence was demonstrated, emphasizing that longer oncological surveillance is necessary to determine long-term benefit.

After the surgery, the patient spent four days in the intensive care unit (ICU), where treatment focused on managing postoperative respiratory failure. According to the Clavien–Dindo classification, the complication is classified as grade II.

The total length of hospital stay was 13 days.

## 3. Discussion

We herein present a case of extensive chest wall metastasis from rectal adenocarcinoma managed with radical resection, complex chest wall reconstruction, and adjunctive HITHOC. Chest wall metastases from colorectal cancer are exceedingly rare and have been reported almost exclusively as isolated case reports [4,5,10,12,13,14,15]. While more than half of patients with colorectal cancer develop liver metastases during the course of the disease, involvement of the chest wall remains exceptional [4,5,10,12,13,14,15,18]. Our case is further distinguished by the extraordinary size of the lesion (25 × 18 × 16 cm), which, to our knowledge, represents the largest chest wall metastasis from rectal adenocarcinoma described in the literature, to date.

Such rare metastases pose unique challenges. First, achieving oncologic radicality (complete resection with negative margins) or sufficient cytoreduction is difficult when a tumour involves multiple thoracic structures (pleura, lung parenchyma, ribs, overlying soft tissue) [19]. Second, reconstructing the resulting defect demands advanced techniques: skeletal stabilization, scaffold or mesh repair, sometimes prosthetics or metal plating, and preservation of chest wall mechanics [20]. For oncological and thoracic surgeons radical resection remains the primary objective, however in certain circumstances it becomes necessary to undertake a procedure aimed at saving life or preventing life-threatening complications [21].

By choosing this form of therapy, both we and our patient were aware that complete remission might not be achievable. Nevertheless, the main goals were short-term local disease control and symptomatic improvement. In this patient, removal of the large chest wall mass resulted in meaningful improvement in comfort and daily functioning.

Our patient initially presented with potentially resectable metastatic disease; therefore, surgical treatment combined with HITHOC and systemic therapy was considered.

Despite ongoing treatment, the tumour demonstrated local progression. Importantly, reassessment with PET-CT did not reveal any additional distant metastases. At our centre, following detailed multidisciplinary evaluation and careful review of imaging studies, the lesion was considered technically resectable with an acceptable perioperative risk. The absence of new distant disease and the patient’s preserved performance status supported an aggressive surgical approach.

Although data from pulmonary and hepatic metastasectomy cannot be directly extrapolated to chest wall metastases, they provide general context for the role of local treatment in metastatic colorectal cancer, with the lungs representing the second most common site of metastases, but isolated pulmonary metastases are relatively rare, accounting for less than 12% of cases [22]. Available evidence suggests that metastasectomy may confer a survival benefit in selected patients [23,24]. This is supported by prospective data, including the Finnish RAXO study, which demonstrated improved outcomes (OS) in patients undergoing resection compared with systemic therapy alone [25]. However, these findings are not directly applicable to massive chest wall metastases, for which survival data remain lacking.

Furthermore, our case illustrates a potential locoregional therapeutic strategy for extensive pleural and chest wall metastatic disease in a highly selected patient, highlighting the importance of a multidisciplinary approach. In comparison with previously reported cases, our management combined radical macroscopic cytoreduction, en bloc chest wall resection with reconstruction, and HITHOC as part of an exploratory locoregional treatment strategy [4,5,6,7,8,9,10,11,12,13,14,15]. This approach should be considered exploratory, as the current evidence for HITHOC is derived mainly from malignant pleural mesothelioma and thymic tumours, while data for secondary pleural metastases from extrathoracic primaries remain limited and largely retrospective [26,27,28,29]. Moreover, there is no standardized HITHOC protocol regarding patient selection, drug choice, temperature, duration, or integration with systemic therapy [30].

When planning the procedure, careful consideration had to be given to chest wall reconstruction. Several options are available, including synthetic meshes, rigid prosthetic implants, and biological grafts, with the choice depending largely on the extent of rib resection, as each method provides a different degree of chest wall stability and mobility [31]. Complete stabilisation, particularly in the early postoperative period, is not always achievable. Chest wall resections are associated with considerable morbidity, with respiratory failure reported in up to 27% of patients [32]. Predisposing factors for respiratory complications include a greater number of resected ribs and concomitant pulmonary lobectomy. Clear correlations between specific prosthetic materials and postoperative complications, however, remain inconclusive [33].

For colorectal cancer, the rationale for our approach was extrapolated from CRS–HIPEC data in peritoneal metastases, where locoregional treatment after maximal cytoreduction is used in selected patients, although the exact benefit of HIPEC remains controversial and the procedure is still protocol-dependent [34,35]. Thus, HITHOC in our patient was not a standard evidence-based treatment for pleural colorectal metastases, but an individualized adjunct intended to improve local control after extensive tumour resection and chest wall reconstruction in a life-threatening thoracic presentation. Notably, no prospective trials on HITHOC for pleural or chest wall metastases from colorectal carcinoma were identified, highlighting both the rarity of this condition and the investigational nature of our approach [36]. Ongoing HITHOC studies primarily concern pleural mesothelioma, while HIPEC trials in colorectal cancer continue to focus on peritoneal disease rather than thoracic involvement [37,38].

HITHOC has been performed for more than 20 years; however, its potential remains insufficiently recognised [39]. It has not been included in clinical guidelines to date, even for malignant pleural mesothelioma or oligometastatic CRC [40,41]. The method has been applied in selected cases of malignant pleural effusion in M1a NSCLC and pleural involvement of thymoma [27,40,41,42,43]. A significant advancement in the safety of the procedure was achieved recently, when it was demonstrated that appropriate nephroprotection reduces renal complications following HITHOC [44]. The use of HITHOC for locoregional disease remains a novel approach. In our case, it was applied as a complementary strategy to reduce microscopic residual disease within the pleural cavity. Emerging evidence suggests that HITHOC may reduce pleural recurrence and prolong PFS in patients with pleural metastatic colorectal cancer. For instance, Markowiak et al. reported that the addition of HITHOC following cytoreductive surgery was associated with median PFS exceeding 12 months in patients with pleural metastatic disease [39]. However, current evidence remains limited and heterogeneous. Available data suggest that cytoreductive surgery combined with HITHOC may be feasible in carefully selected patients with secondary pleural malignancies and may contribute to improved local disease control [29]. This is supported by a 2023 German multicentre series, in which macroscopic complete resection was achieved in 90% of cases, with a median overall survival of 39 months, supporting the concept that maximal cytoreduction remains the key prerequisite for considering adjunct intrathoracic chemoperfusion [26].

In our patient, the combination of surgical resection, chest wall reconstruction, and HITHOC demonstrates the strength of a comprehensive approach in the management of disseminated disease. The procedure required close collaboration between surgical oncology, thoracic surgery, anaesthesiology, and clinical oncology [29,39]. A major strength of our treatment is the radical removal of lesions, offering a potential for improved disease control [45]. Although the procedure enabled complete resection of the metastatic lesion, it was associated with clinically significant postoperative morbidity, including severe chest pain, persistent tachycardia, and respiratory failure requiring intensive care. These complications highlight the substantial physiological burden of extensive chest wall resection and should be carefully considered when evaluating the overall risk–benefit balance of such aggressive surgical strategies in patients with metastatic disease [46]. Nevertheless, in carefully selected patients, radical resection may still represent a reasonable option for achieving local disease control.

This approach is associated with significant surgical burden and potential complications, including respiratory failure, infection, and prosthetic-related issues [28]. Chest wall reconstruction may additionally result in paradoxical breathing, chronic pain, and reduced mobility, with infection being the most common complication [33]. Importantly, HITHOC remains insufficiently studied in colorectal cancer, with no reliable data on long-term oncological outcomes. Therefore, although local disease control may be achieved, its impact on overall survival and distant recurrence remains uncertain. Importantly, the contribution of HITHOC to the observed short-term outcome cannot be determined in a single case. Moreover, such an extensive multimodal approach is applicable only in carefully selected patients, particularly given the risks and contraindications associated with HITHOC, including severe renal impairment and significant cardiac disease [39,47,48]. As a single-case report with limited follow-up, this study cannot establish causal relationships or confirm long-term benefit, but rather demonstrates the feasibility of extensive chest wall resection combined with HITHOC as part of an aggressive multidisciplinary treatment strategy in a highly selected patient, illustrating a potential strategy for local disease control in this rare clinical scenario.

Given the rarity of chest wall metastases, future efforts should focus on multicentre data collection, prospective evaluation of HITHOC, and early multidisciplinary management to optimize outcomes.

## 4. Conclusions

This case demonstrates that aggressive multimodal management, including radical resection, chest wall reconstruction, and adjunctive intrathoracic chemotherapy, may be associated with short-term local disease control in selected patients with otherwise inoperable chest wall metastases from colorectal cancer. Successful treatment in our patient was enabled by early multidisciplinary evaluation and coordinated decision-making. In the present case, local control was most likely achieved primarily through complete surgical resection, while the additional contribution of HITHOC remains uncertain. This approach may expand therapeutic options and allow continuation of systemic therapy in highly selected cases. However, its oncological benefit remains uncertain, and further evidence from larger clinical series is required to define its role.

## Data Availability

The data are not publicly available due to privacy or ethical restrictions.
